# Protocol to study inflammasome activation in induced pluripotent stem cell-derived macrophages

**DOI:** 10.1016/j.xpro.2026.104747

**Published:** 2026-07-28

**Authors:** Chloe M. McKee, Melanie Cranston, Emma C. McKay, Mohammad Arefian, Ben C. Collins, Rebecca C. Coll

**Affiliations:** 1 The Wellcome-Wolfson Institute for Experimental Medicine, Queen’s University of Belfast, Belfast, UK; 2 School of Biological Sciences, Queen’s University of Belfast, Belfast, UK

**Keywords:** Cell culture, Flow Cytometry, Immunology, Stem Cells, Cell Differentiation, Mass Spectrometry

## Abstract

Macrophages are widely used to study inflammasome activity, but current *in vitro* macrophage models have various limitations. Here, we describe a protocol to differentiate induced pluripotent stem cell (iPSC)-derived macrophages (iMacs) and to characterize these cells via flow cytometry, phagocytosis assays, and whole-cell proteomics. We then describe how to activate a range of different inflammasomes within these cells and assess the inflammasome response through measurement of pyroptosis, cytokine release, ASC speck formation, and protein processing via western blotting.

For complete details on the use and execution of this protocol, please refer to McKee et al.^1^

## Highlights

• Comprehensive and scalable protocol for differentiating iPSC-derived macrophages
• Steps to characterize macrophage marker expression and phagocytosis
• Whole-cell proteomic approach to examine iMac responses to inflammatory stimuli
• iMacs are a physiologically relevant model for human inflammasome studies

## Abstract (editor highlights)

Publisher’s note: Undertaking any experimental protocol requires adherence to local institutional guidelines for laboratory safety and ethics.

## Graphical abstract

**Figure:**
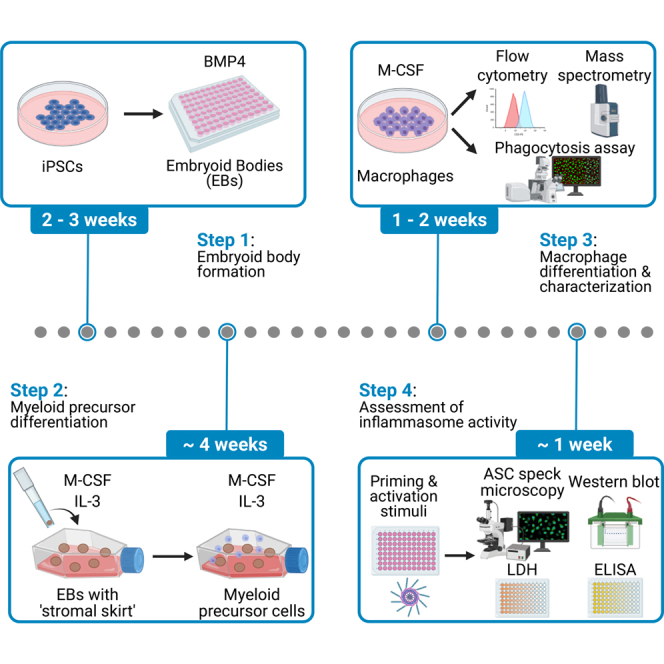

## Before you begin

Here we describe the specific steps involved in differentiating iMacs, characterization of the cells through flow cytometry, phagocytosis assays and global proteomics, and the activation of the NLRP3, NAIP/NLRC4, NLRP1/CARD8, and non-canonical inflammasomes within these cells.

### Innovation

Inflammasome sensors such as NLRP3 are predominantly expressed by macrophages and other myeloid cells.^2^ However, current *in vitro* macrophage models, including mouse bone marrow-derived macrophages, monocytic cancer cell lines such as THP-1 cells, and primary human monocyte-derived macrophages, have various limitations with regards to studying inflammasome biology.^1^ iMacs offer new opportunities to study inflammasome activation as they offer a continuous and scalable supply of cells that can be genetically modified and patient specific.^3^^,^^4^ However, to date very few studies have used iMacs to study inflammasome signaling and there has been no in-depth characterization of the different inflammasome responses in these cells.

This protocol establishes a comprehensive reference framework for studying NLRP3 and other inflammasomes in iMacs. We detail how to assess inflammasome activity through measuring pyroptosis, cytokine release, ASC speck formation, and protein processing via western blotting. In addition, we describe how to perform whole cell proteomic analysis of iMacs to assess the expression of inflammasome- and inflammation-related proteins in response to pattern recognition receptor (PRR) stimulation. This enables unbiased characterization of the inflammatory state of iMacs and provides a global resource describing how PRR stimulation alters inflammasome-related protein networks. We demonstrate that iMacs are a physiologically relevant and highly tractable model to study human inflammasome signaling and regulation. Therefore, the expectation is that iMacs will be of significant use in future inflammasome studies. In particular, as iMacs can be genetically modified and patient-specific, this protocol provides a platform for mechanistic studies and investigating autoinflammatory diseases, immunodeficiencies, and rare inflammasome-opathies.

## Key resources table

**Table:** 

REAGENT or RESOURCE	SOURCE	IDENTIFIER
**Antibodies**		

Anti-human CD14, FITC (1:500)	BioRad	MCA1568A488; RRID:AB_3100368
IgG2a Negative control, FITC (1:500)	BioRad	MCA929A488; RRID:AB_324436
Anti-human CD16, PE/Cyanine7 (1:50)	Biolegend	302016; RRID:AB_314216
IgG1, κ Isotype Control, PE/Cyanine7 (1:50)	Biolegend	400126; RRID:AB_326448
Anti-human CD44, APC (1:400)	BioRad	MCA89A647T; RRID:AB_1102041
IgG2a Negative Control, APC (1:400)	BioRad	MCA929A647T; RRID:AB_322306
Anti-human CD64, PE (1:100)	BioRad	MCA756PE; RRID:AB_321800
IgG1 Negative Control, PE (1:100)	BioRad	MCA928PE; RRID:AB_322263
Anti-human CD68, PE (1:50)	BioRad	MCA6014PE; RRID:AB_3101254
IgG2b Negative Control, PE (1:50)	BioRad	MCA691PE; RRID:AB_322277
Fixable Viability Dye eFluor (1:500)	eBioscience	65-0866-14
LysoTracker Red DND-99 (1:2000)	ThermoFisher	L7528
Anti-β-actin (1:5000)	Sigma-Aldrich	A2228; RRID:AB_476697
Anti-caspase-1 (D7F10) (1:1000)	Cell Signaling Technology	3866; RRID:AB_2069051
Anti-cleaved caspase-1 (1:1000)	Cell Signaling Technology	4199S; RRID:AB_1903916
Anti-GSDMD N-terminal (1:1000)	Abcam	ab215203; RRID:AB_2916166
Anti-GSDMD C-terminal (1:1000)	Abcam	ab210070; RRID:AB_2893325
Anti-IL-1β (1:1000)	R&D Systems	AF-401-NA; RRID:AB_416684
Anti-NLRP3 (Cryo-2) (1:1000)	Adipogen	AG-20B-0014; RRID:AB_2490202
Anti-NLRP3 (D4D8T) (1:1000)	Cell Signaling Technology	15101; RRID:AB_2722591
HRP- Anti-Rabbit IgG (1:5000)	Jackson Immunoresearch	111-035-003; RRID:AB_2313567
HRP- Anti-Goat IgG (1:5000)	Jackson Immunoresearch	705-035-147; RRID:AB_2313587
HRP- Anti-Mouse IgG (1:5000)	Jackson Immunoresearch	515-035-003; RRID:AB_2340295
Anti-ASC (1:50)	BioLegend	676502; RRID:AB_2565643
Alexa Fluor™ 488 Donkey anti-mouse (1:800)	Invitrogen	A21202; RRID:AB_141607

**Chemicals, peptides, and recombinant proteins**		

Vitronectin	ThermoFisher	A14700
StemFlex medium	ThermoFisher	A3349401
Rho-kinase (ROCK) inhibitor Y- 27632 Dihydrochloride	Merck	Y0503
UltraPure™ EDTA, pH 8.0	ThermoFisher	15575020
DPBS (10X), no calcium, no magnesium	ThermoFisher	14200075
KnockOut Serum Replacement	ThermoFisher	10828010
Bone Morphogenetic Protein 4 (BMP-4)	R&D Systems	314-BP010
Gelatine	Sigma-Aldrich	G1890
Water for embryo transfer	Sigma-Aldrich	W1503
X-VIVO 15 Serum Free Medium	Lonza	BE02-060F
GlutaMAX	ThermoFisher	35050061
Penicillin-streptomycin	ThermoFisher	15140122
HumanKine recombinant human M-CSF protein	Proteintech	HZ-1192
Human IL-3 Recombinant Protein	Peprotech	200-03-250UG
Lidocaine monohydrate	Sigma-Aldrich	L5647-100g
RMPI 1640	ThermoFisher	21870076
Fetal bovine serum (FBS)	ThermoFisher	26140079
Ultrapure LPS from E. coli K12	InvivoGen	tlrl-peklps
Triacylated lipopeptide Pam3CSK4	InvivoGen	tlrl-pms
Nigericin sodium salt	AdipoGen	AGCN2-0020-M005
Adenosine 5′-Triphosphate (ATP) disodium salt	Merck Milipore	1191-1GM
Lipofectamine LTX Reagent with PLUS Reagent	ThermoFisher	A12621
Lfn-BsaL (a fusion protein of the N-terminal domain of the Bacillus anthracis lethal factor and BsaL needle protein from *Burkholderia Pseudomallei*)	InvivoGen	tlrl-ndl
Anthrax Protective Antigen (PA), Recombinant from *Bacillus anthracis*	Quadratech Diagnostics	LL-171E
Talabostat mesylate	R&D Systems	3719/10
Anisomycin from Streptomyces griseolus	Merck	A9789
Human TruStain FXX	Biolegend	422302
eBioscience™ Intracellular Fixation & Permeabilization Buffer Set	ThermoFisher	88-8824-00
OptiMEM	ThermoFisher	31985070
Latex amine-modified polystyrene, fluorescent yellow-green beads	Sigma-Aldrich	L1030-1ML
4% paraformaldehyde (PFA)	Merck Life Science	818715
Triton X-100	Sigma-Aldrich	T9284-500ML
Bovine serum albumin (BSA)	Sigma-Aldrich	A7906-100G
Vectashield Antifade Mounting Medium with DAPI	Vector Laboratories	H-1299
Phosphate-buffered saline (PBS)	Sigma-Aldrich	P4417-50TAB
Tween®20	Sigma-Aldrich	P7949-500ML
BaseMuncher	Expedeon	BM0025
NuPAGE LDS sample buffer	Invitrogen	NP0007
Dithiothreitol (DTT)	Merck Life Science	20-265
4–20% Mini-PROTEAN® TGX™ Precast Protein Gels, 10-well	BioRad	4561093
Tris	Sigma-Aldrich	T6066-1KG
Glycine	Melford	56406
Sodium dodecyl sulfate (SDS)	Merck	L3771
Precision Plus Protein Kaleidoscope Standard	BioRad	1610375EDU
Nitrocellulose membrane	BioRad	1620115
Filter paper	BioRad	1704085
Trans-Blot Turbo 5x Transfer Buffer	BioRad	10026938
Ethanol	Fisher Scientific	16685992
Sodium chloride	Fisher Scientific	S/3120/65
Fat-free powdered milk	Marvel	N/A
Clarity ECL	Biorad	170-5061
SuperSignal™ West Atto	Thermo Fisher	A38555
Hydrogen peroxide	Sigma-Aldrich	16911-1L-F
Sodium deoxycholate	Sigma-Aldrich	D6750-100G
Tris (2-carboxyethyl) phosphine hydrochloride (TCEP)	Sigma-Aldrich	C4706-2G
2-Chloroacetamide ≥98.0% (HPLC)	Sigma-Aldrich	22790-250G-F
MagReSyn Hydroxyl beads	ReSyn Biosciences	MR-HYX100
Acetonitrile (ACN)	Sigma-Aldrich	302031-100ml
Triethylammonium bicarbonate buffer (TEAB)	Sigma-Aldrich	T7408
Lys-C	Thermo Fisher	90397
Trypsin	Pierce	90058

**Critical commercial assays**		

Cytotoxicity Detection Kit Roche	Sigma-Aldrich	11644793001
LDH-Glo Cytotoxicity Assay	Promega	J2381
Human IL-1 beta/IL-1F2 DuoSet ELISA	R&D Systems	DY201
Human TNF-alpha DuoSet ELISA	R&D Systems	DY210
Human IL-18 ELISA kit	ThermoFisher	BMS267-2MST
Pierce™ Rapid Gold BCA Protein Assay Kit	ThermoFisher	A53226

**Deposited data**		

Proteomics data set	PRIDE website	https://www.ebi.ac.uk/pride/login: Project accession: PXD068076 Token: ilXveQUXDATv

**Experimental models: Cell lines**		

Human induced pluripotent stem cell line	Wellcome Sanger Institute	HPSI0114i-kolf_2-C1

**Software and algorithms**		

Biorender	Biorender Software	https://biorender.com/
Excel	Microsoft Office	https://www.office.com/
Graphpad Prism 10	GraphPad Software	https://www.graphpad.com/
Affinity designer	Serif	https://affinity.serif.com
FlowJo	BD Biosciences	https://www.flowjo.com/
Leica LAS X	Leica LAS X software	https://www.leica-microsystems.com/products/microscope-software/p/leica-las-x-ls/
Fiji Image J	Image J Software	https://imagej.net/software/fiji/
Spectronaut	Biognosys	https://biognosys.com/software/spectronaut/
RStudio	RStudio	https://posit.co/download/rstudio-desktop/

**Other**		

10 cm non-treated cell culture dish	Sarstedt	83.3900
6 well plate	Sarstedt	83.3920
24 well plate	Sarstedt	83.3922
96 well plate	Sarstedt	83.3924
T175 flask	Sarstedt	83.3912.002
Low-adherent, U-bottom 96 well plate	Aquilant	650970
Uncoated ELISA plate	VWR International	735-0199
Eppendorf microcentrifuge tubes (1.5 mL)	Sarstedt	72.690.001
Centrifuge tubes (15 mL)	Sarstedt	62.554.502
Centrifuge tubes (50 mL)	Sarstedt	62.547.254
Pipette tips (10 μL)	Sarstedt	70.1130
Pipette tips (200 μL)	Sarstedt	70.3030
Pipette tips (1000 μL)	Sarstedt	70.3050
Serological Pipette 25 mL	Sarstedt	86.1685.001
Serological Pipette 10 mL	Sarstedt	86.1254.001
Serological Pipette 5 mL	Sarstedt	86.1253.001
Tube 50 mL	Sarstedt	62.547.254
Tube 15 mL	Sarstedt	62.554.502
Cell Scraper	Sarstedt	83.3951
Coverslips	VWR International	631-0149
Evotips	Evosep	EV2011
Laminar flow tissue culture hood	Contained Air Solutions	N/A
Tissue culture incubator	Panasonic Healthcare Co	N/A
FACS Canto II cytometer	Becton Dickenson	N/A
Stellaris-5 confocal microscope	Leica Microsystems	N/A
FLUOStar Omega plate reader	BMG Biotech	https://www.bmglabtech.com/en/fluostar-omega/
Mini-PROTEAN Tetra Vertical Electrophoresis Cell	BioRad	1658004
Trans-Blot® Turbo™ Transfer System	BioRad	1704150EDU
G:BOX	SYNGENE	N/A
DM5500 fluorescent microscope	Leica Microsystems	N/A
TimsTOF HT quadrupole time-of-flight mass spectrometer	Bruker	N/A

## Materials and equipment

**iPSC culture medium** – 450 mL basal StemFlex medium & 50 mL supplement (Store at 4°C and use for the next 2–3 months or aliquot and freeze at −20°C for up to 6 months).

***Alternatives:*** Essential 8 Medium (ThermoFisher, Cat #: A1517001) can be used as iPSC culture medium instead of StemFlex Medium.

**Table:** Myeloid precursor base medium

Reagent	Final concentration	Volume/Mass
X-VIVO 15 Serum Free Medium	1 X	492.5 ml
GlutaMAX	2 mM	5 ml
Penicillin-streptomycin	100 IU/ml	2.5 ml
β-mercaptoethanol	0.1 mM	3.5 μl
Total	N/A	500 ml

Store at 4°C and use for the next 2–3 months.

**CRITICAL:** β-mercaptoethanol can be toxic if ingested, and fatal if inhaled or absorbed through the skin. Always use within the tissue culture fume hood.

**Table:** iMac medium

Reagent	Final concentration	Volume/Mass
RMPI 1640	1 X	442.5 ml
FBS	10 %	50 ml
GlutaMAX	2 mM	5 ml
Penicillin-streptomycin	100 IU/ml	2.5 ml
Total	N/A	500 ml

Store at 4°C and use for the next 2–3 months.

**Table:** **S**DC lysis buffer

Reagent	Final concentration	Volume/Mass
Sodium deoxycholate BioXtra, ≥98.0% (dry matter, NT)	1%	50 μl of 10% SDC (0.05 g:0.5 ml water)
Tris (2-carboxyethyl) phosphine hydrochloride (TCEP)	10 mM	28.66 μl of 5% TCEP (0.00143325 g) (for 5%- 0.002 g:40 μl)
2-Chloroacetamide ≥98.0% (CAA)	40 mM	37.40 μl of 5% CAA (0.0018702 g) (for 5%- 0.002 g:40 μl)
Tris pH 8.5	100 mM	100 μl 0.5 M
dH2O	N/A	316.97 μl
Total	N/A	533 μl

Make up fresh for each use.

**Table:** **P**BS-Tween

Reagent	Final concentration	Volume/Mass
PBS	10 X	50 tablets
Tween®20	0.5%	5 ml
dH2O	N/A	1 L
Total	N/A	1 L

10X, ELISA wash buffer, store at ∼20°C and use for the next 6 – 12 months.

**Table:** **R**unning buffer

Reagent	Final concentration	Volume/Mass
Tris	250 mM	30.36 g
Glycine	1.5 M	144.13 g
Sodium dodecyl sulfate	1%	10 g
dH2O	N/A	1 L
Total	N/A	1 L

10X, store at ∼20°C and use for the next 6 – 12 months.

**Table:** **T**ransfer buffer

Reagent	Final concentration	Volume/Mass
Trans-Blot Turbo 5x Transfer Buffer	1X	100 ml
Ethanol	N/A	100 ml
dH2O	N/A	300 ml
Total	N/A	500 ml

Store at 4°C and use for the next 3 – 6 months.

**Table:** TBS-Tween

Reagent	Final concentration	Volume/Mass
Tris Hydrogen chloride pH 8	100 mM	100 ml (121.44 g in 1 L dH2O, pH to 8 with 3 M Hydrogen chloride)
Sodium chloride	1.5 M	87.66 g
Tween®20	0.5%	5 ml
dH2O	N/A	895 ml
Total	N/A	1 L

10X, Western wash buffer, store at ∼20°C and use for the next 6 – 12 months.

## Step-by-step method details

### iPSC culture

**Timing: 1–2 weeks**

This step cultures and passages iPSCs to allow for later formation of embryoid bodies (EBs).

1. Thawing and culture of iPSCs.
  a. Coat plates with vitronectin to allow iPSCs to attach to the plate surface.
    i. Dilute 0.5 mg/mL vitronectin stock 1:100 in DBPS.
    ii. Add 1 mL 5 μg/mL vitronectin in DPBS into two wells of a 6-well tissue culture plate.
      ***Note:*** Ensure that the vitronectin covers the entire surface of the wells.
  b. Incubate the plate(s) for at least 2 h at ∼20°C or up to 24 h at 4°C after sealing tightly with Parafilm.
  c. Warm 9 mL StemFlex medium in a 15 mL tube with 1 μM Rho-associated protein kinase (ROCK) inhibitor at 37°C.
    ***Note:*** ROCK inhibitor is used to prevent apoptosis and allow recovery of iPSCs following freezing or passaging. If it is not included in the StemFlex medium, the iPSCs will not attach to the vitronectin-coated surface and this will result in cell death. 1 μM is used for initially thawing iPSCs but 10 μM is required when plating cells.
  d. Thaw a vial of iPSCs and transfer gently into the 15 mL tube.
  e. Centrifuge the tube at 300 × g for 3 min at ∼20°C.
  f. Resuspend the cell pellet gently using the desired volume of StemFlex medium supplemented with 10 μM ROCK inhibitor.
  g. Remove the vitronectin from the wells of the 6-well plate.
  h. Add resuspended iPSCs to two wells of the 6-well plate.
    ***Note:*** The contents of one frozen vial can be distributed into 2 wells of a 6-well plate (25% of the cells into one well and 75% of cells into another well in a total volume of 1 mL StemFlex medium).
  i. Place the plates in a tissue culture incubator at 37°C with 5% CO_2_ in a humidified atmosphere.
  j. Swirl the plates gently to ensure the colonies are evenly distributed across the surface of the plates.
  k. Leave the plates undisturbed for up to 24 h to allow the colonies to attach.
  l. After 24 h, remove all media from the plates to discard unattached colonies and dead cells.
  m. Add 2–3 mL fresh StemFlex media (without ROCK inhibitor) for each well of the 6-well plate by pipetting slowly onto the side of the well to avoid disturbing the iPSC colonies.
    ***Note:*** Although ROCK inhibitor is necessary when initially plating iPSCs to prevent apoptosis, it must be removed after 24 h to allow iPSC colony expansion.
  n. Replace the medium daily with fresh StemFlex medium (without ROCK inhibitor) until cells reach ∼70–80% confluency.
2. Passaging iPSCs.
  a. Coat 100 mm plates with 6 mL 5 μg/mL vitronectin as described in section 1a.
  b. Remove the media from the cells and gently wash the plate twice with DPBS.
  c. Add 0.5 mM PBS-EDTA to cover the entire surface of the plates (1 mL for each well of a 6-well plate or 5 mL for a 100 mm dish).
  d. Return the plate to the incubator for 4 min.
  e. Check the cells using the microscope to confirm that the morphology of the colonies has changed and that they are beginning to detach.
    ***Note:*** Do not leave the iPSCs in PBS-EDTA for too long as the colonies will fully detach from the plates and will break up into single cells, leading to cell death.
  f. Remove PBS-EDTA solution gently without disturbing the iPSC colonies.
  g. In the 6 well plate, add 1 mL StemFlex directly onto the base of the well to wash off the cells but do not pipette up and down.
  h. Repeat five times to add a total of 6 mL StemFlex while turning the plate to ensure the cells are washed off the entire surface of the well.
  i. Use a 10 mL pipette to gently transfer the cells.
    ***Note:*** If passaging cells from a 100 mm dish, add fresh StemFlex media and use a 10 mL pipette to gently pipette up and down to dislodge the colonies from the surface without breaking them into a single cell suspension.
  j. Transfer the media with the detached iPSC colonies into a 15 mL tube using a 10 mL pipette.
  k. Centrifuge at 300 × g for 3 min at ∼20°C.
  l. Gently resuspend the colonies in 10 mL of fresh StemFlex media supplemented with 10 μM ROCK inhibitor.
  m. Add the colonies in a desired splitting ratio into a fresh vitronectin-coated 100 mm plate with 10 mL StemFlex media supplemented with 10 μM ROCK inhibitor.
    ***Note:*** Replate 1 well of a 6 well plate directly onto 1 100 mm plate and split confluent 100 mm plates between 1:5 and 1:10.
  n. After 24 h, replace with fresh StemFlex medium (without ROCK inhibitor).
  o. Continue to change medium daily until the cells are ready for the next passage.
3. Freezing iPSCs.
  a. Prepare the freezing medium consisting of 10% DMSO in KnockOut Serum Replacement (1 mL DMSO in 9 mL KnockOut Serum Replacement).
  b. Detach iPSC colonies as described in section 2b – k.
  c. Resuspend the cell pellet in freezing medium.
  d. Add 1 mL cell suspension into each cryovial.
    ***Note:*** Freeze ∼10 cryovials from each ∼70–80% confluent 100 mm plate, approximately 1x10^6^ cells per vial.
  e. Place the cryovials immediately into a Mr Frosty container and leave for up to 24 h at −80°C.
  f. Transfer the cryovials into a −150°C freezer or liquid nitrogen for long-term storage.
    ***Note:*** It is important to carefully select the iPSC line to use as the differentiation potential of iPSC lines can vary for different cell types, with some showing greater efficiency in differentiating into specific cells. This is influenced by transcriptional and epigenetic variation between donors.^5^ The kolf-2 iPSC line has a high level of efficiency for differentiating into iMacs while we found the HPSI0314i-hoik_1 (WTSIi026-A) cell line to be unsuccessful in differentiating.
    **CRITICAL:** Daily media changes are essential as iPSCs may spontaneously differentiate into fibroblast-like cells without the addition of fresh medium containing cytokines and growth factors that maintain the cells in a pluripotent state.
    **CRITICAL:** Human iPSCs are delicate and grow into individual colonies. It is important that when resuspending these cells, they are not broken down into a single cell suspension but instead are pipetted gently to maintain the cells in small clumps. In addition, cells should be centrifuged slowly (maximum speed of 300 × *g*) and with shorter duration where possible.
    **CRITICAL:** Always pre-warm media and other reagents to ∼20°C before adding to iPSCs as the cells are sensitive to temperature changes.

### Myeloid precursor generation and macrophage differentiation

**Timing: 4–5 weeks**

This protocol first describes formation of EBs from iPSCs using centrifugation and low adherent, U-bottom 96 well plates. It then uses M-CSF and IL-3 to drive the EBs towards a myeloid lineage, with the subsequent production of myeloid precursor cells that are further differentiated into macrophages with M-CSF.

**Figure 1:**
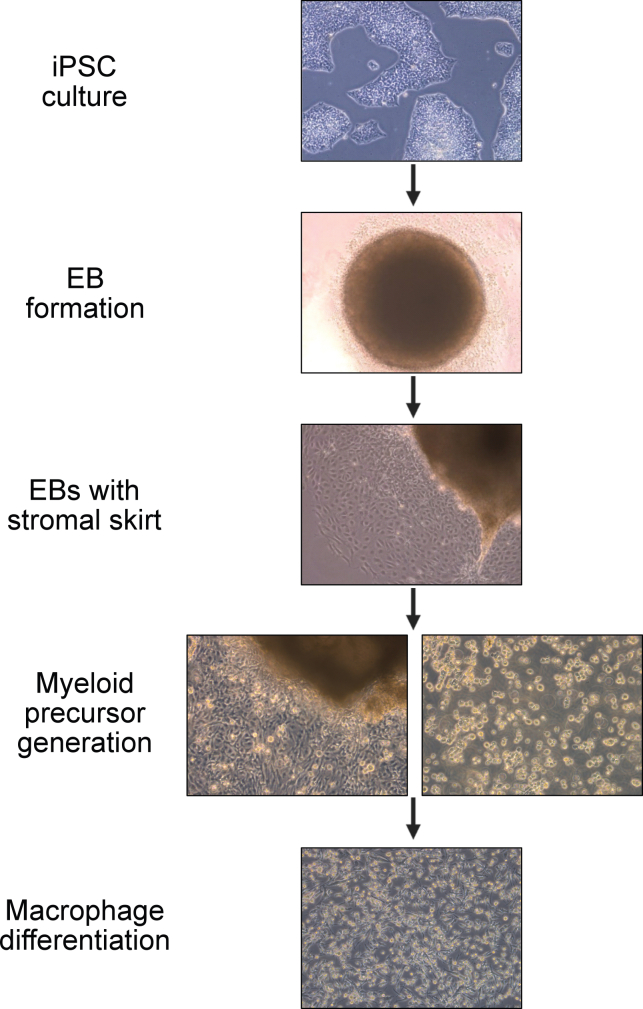
iPSC-macrophage differentiation iPSCs are cultured on vitronectin-coated plates to form colonies, then harvested and seeded into U-bottomed low-adherent 96 well plates in the presence of BMP-4 to stimulate the formation of embryoid bodies (EBs). EBs are transferred to gelatine-coated flasks and adhere to the surface, forming a layer of stromal cells. After 3-4 weeks of differentiation with M-CSF and IL-3, EBs begin to produce myeloid precursor cells which can be seen floating in the medium. The precursors are harvested and plated out with M-CSF to differentiate into macrophages (iMacs).

4. Embryoid body formation.
  a. Harvest cells with 0.5 mM PBS-EDTA as described in section 2b – k.
  b. Count a sample of these cells using a hemocytometer and Trypan blue solution.
    ***Note:*** iPSCs are difficult to resuspend to a single cell suspension, therefore there may still be small clumps of cells when counting.
  c. Spin cells at 300 × *g* for 3 min at ∼20°C.
  d. Resuspend in StemFlex media supplemented with Bone Morphogenetic Protein 4 (BMP-4, 50 ng/mL) and ROCK inhibitor (10 μM) to a final concentration of 500,000 cells/mL.
  e. Seed cells into a low adherent, U-bottom 96 well plate at 50,000 cells per well, adding a final volume of 100 μL.
    ***Note:*** The optimal number of cells to use for EB formation may require adjustment for each iPSC line used. In our hands, 50,000 cells per well formed smaller but more compact and spherical EBs compared to 100,000 cells per well and subsequently generated higher yields of myeloid precursor cells.
  f. Wrap the plates in parafilm and spin at 300 × *g* for 3 min at ∼20°C.
  g. Remove parafilm and leave the plate in the incubator for 5 days, beginning day 0.
  h. Feed the EBs on day 2 – replace 50 μL media with 50 μL fresh StemFlex media with BMP-4 without disturbing the EBs.
    ***Note:*** Alternatively, to complete a 75% media change, remove 50 μL media, replace with 50 μL fresh media, remove 50 μL again and replace with 50 μL fresh media.
  i. Repeat on day 4.
5. Myeloid precursor generation.
  a. Add 50 mg gelatine to 50 mL water for embryo transfer and heat to 56^o^C for 30-45 min in a water bath.
  b. Filter sterilize with 0.2 μm filter.
  c. Add 12 mL gelatine to each T75 flask or 25 mL to each T175 flask for 2 h at ∼20^o^C or store at 4^o^C for up to 24 h after wrapping the lids in parafilm.
  d. On day 5, use 1 mL pipette tips to pool EBs in a 50 mL tube in the required numbers and aspirate most of the supernatant.
    ***Note:*** Cut the tips from 1 mL pipette tips using sterile scissors to aspirate the EBs from the 96 well plate without disturbing them.
  e. Remove the gelatine from the flasks and add myeloid precursor base medium (X-VIVO 15 serum-free medium; 2 mM GlutaMAX, 100 IU/mL penicillin-streptomycin, 0.1 mM β-mercaptoethanol with 50 ng/mL M-CSF and 25 ng/mL IL-3).
  f. Add 5 mL myeloid precursor base medium to the EBs and transfer to a gelatine-coated flask.
  g. Gently rock the flasks to ensure the EBs are evenly distributed across the surface.
  h. Leave the flasks undisturbed in a tissue culture incubator at 37°C with 5% CO_2_ for one week.
    ***Note:*** Generally, we add 30 EBs per T175 flask in 30 mL medium or 20 EBs per T75 flask in 20 mL medium. The number of EBs to add per flask may require optimization for each iPSC line.
  i. On day 7 of EB culture, top up the flasks with another 10 mL of myeloid precursor base media supplemented with IL-3 and M-CSF.
  j. Leave the EBs undisturbed for an additional 7 days.
    ***Note:*** After one week, most of the EBs will attach to the gelatine-coated surface of the flasks and begin to form a layer of stromal cells around the EB termed the ‘stromal skirt’.
  k. To change the medium, collect all medium from the EB flasks using a 25 mL pipette avoiding the EBs where possible.
    ***Note:*** Initially all EBs will be adhered to the flasks but over the weeks in culture, some will begin to detach and float in the medium. If any floating EBs are collected into the pipette, let them sink to the bottom of the pipette and then expel back into the flask.
  l. Add fresh cytokine-supplemented myeloid precursor base media.
  m. Repeat media change every 7 days, topping up the flasks with an additional 10 mL medium after 4-5 days until EBs stop producing myeloid precursor cells.
    ***Note:*** After around 3–4 weeks of culture in the gelatine-coated flasks, the EBs will begin to produce myeloid precursor cells that float in suspension in the culture and look like large blast-like cells. Myeloid precursors can be harvested every 7 days. This is a continuous culture that can be maintained for several months before the number of myeloid precursor cells begins to decline. The EBs will continue to produce precursors as long as cytokine-supplemented fresh medium is added. The duration of myeloid precursor cell production varies with each batch of EBs.
    **CRITICAL:** Do not move the EB flasks at all in the first week of culture to allow the EBs to attach fully to the surface of the flasks. Many of the EBs will detach from the flask surface over time but we have found that the more EBs that initially adhere and form a stromal skirt on the base of the flask, the greater the yield of myeloid precursor cells.
    **CRITICAL:** Always ensure that the EBs are frequently given fresh medium as myeloid precursor production is greatly dependent on maintaining the required concentration of IL-3 and M-CSF and the levels of nutrients.
6. Macrophage differentiation and harvesting.
  a. Remove the medium from the EB flask and filter through a 70 μm cell strainer.
  b. Collect the flow through in 50 mL tubes as it contains the myeloid precursor cells.
  c. Centrifuge at 300 × g for 3 min at ∼20°C.
  d. Resuspend the pellet in macrophage differentiation medium (RPMI 1640; 10% heat-inactivated fetal calf serum; 2 mM L-GlutaMAX; 100 IU/mL penicillin-streptomycin) supplemented with 100 ng/mL M-CSF.
  e. Count cells and plate ∼500,000 cells in a 100 mm tissue culture dish.
  f. Culture the myeloid precursor cells in a 37°C incubator for another 7 days.
  g. Add 4 mL macrophage differentiation medium with M-CSF on day 4 or 5.
    ***Note:*** After 7 days, myeloid precursor cells are fully differentiated into mature macrophages (iMacs) ready for use in experiments.
  h. On day 7, remove medium and wash cells twice with DPBS.
  i. Add lidocaine monohydrate in DPBS to cover the entire surface of the plates.
  j. Return the plate to the incubator for 4 min.
  k. Check under the microscope to ensure the cells are detaching then flush the cells off the plates using RPMI.
  l. Centrifuge the cells for 5 min at 500 × *g* at ∼20°C and resuspend in iMac medium.
  m. Count cells and plate iMacs onto 96-, 24-, and 6-well plates at 0.5x10^5^ in 100 μL, 1x10^5^ in 500 μL, and 1x10^6^ cells in 1 mL per well respectively in iMac medium.
    Images from steps 1 – 6 are shown in Figure 1.

### Characterizing iMacs

**Timing: 1–2 weeks**

This protocol describes how to characterize iMacs by assessing the expression of macrophage markers via flow cytometry, phagocytic activity via confocal microscopy, and inflammasome-related protein expression via proteomics analysis.

7. Flow cytometry.
  a. Harvest and count iMacs as described in section 6h – l.
  b. Resuspend iMacs and add 0.5x10^6^ cells per round bottom flow tube in 1 mL DPBS.
  c. Centrifuge the flow tubes for 5 min at 500 × *g*, ∼20°C.
  d. Discard the supernatant and stain all cells with eBioscience Fixable Viability Dye eFluor 506 at a dilution of 1:500 for 10 min at ∼20°C.
  e. Add 1 mL DPBS and centrifuge cells for 5 min at 500 × *g*, ∼20°C.
  f. Repeat this wash step twice, discarding the supernatant between washes.
  g. Add 100 μL blocking solution (Human TruStain FXX) at a dilution of 1:20 for 30 min in the dark at ∼20°C.
  h. Centrifuge the flow tubes for 5 min at 500 × *g*, ∼20°C and discard the supernatant.
  i. Wash the cells twice with 1 mL DPBS and centrifuge for 5 min at 500 × *g*, ∼20°C. Discard the supernatant between washes.
  j. For intracellular antibodies (CD68);
    i. Resuspend cells in 200 μL fixation buffer and incubate for 30 min at ∼20°C.
    ii. Centrifuge the cells for 3 min at 800 × *g* and discard the supernatant.
    iii. Resuspend the cell pellet in 200 μL permeabilization buffer (diluted 1:10 in dH_2_O from the 10X stock).
    iv. Centrifuge the cells for 5 min at 500 × *g* and discard the supernatant.
    v. Resuspend the cell pellet in 500 μL permeabilization buffer.
    vi. Add the appropriate antibody/isotype control concentration to each tube.
    vii. Incubate for 30 min in the dark at 4°C.
  k. For extracellular antibodies (CD14, CD16, CD44, and CD64);
    i. Add 500 μL DPBS to each tube with the appropriate antibody/isotype concentration.
    ii. Incubate for 30 min in the dark at 4°C.
  l. Centrifuge the flow tubes for 5 min at 500 × *g*, ∼20°C and discard the supernatant.
  m. Wash the cells twice with 1 mL DPBS and centrifuge for 5 min at 500 × *g*, ∼20°C. Discard the supernatant between washes.
  n. Resuspend cells stained for extracellular antibodies in 250 μl DPBS.
  o. Resuspend cells stained for intracellular antibodies in 250 μl permeabilization buffer.
  p. Keep cells on ice.
  q. On the flow cytometer, run an unstained control with each experiment to set up the voltages for the forward scatter (FSC) and side scatter (SSC).
  r. Use the FSC-area (-A) and SSC-A to draw a gate on the scatter plots to exclude cell debris and dead cells.
    ***Note:*** This selected population is used when setting up antibody voltages.
  s. Use isotype controls to set up the voltages for each antibody so that the histogram peak for the isotype control is approximately at 10^2^.
  t. Run each sample on the machine and record 10,000 events.
    ***Note:*** The number of events recorded could be increased to 50,000 if required to lead to better statistics.
  u. In the FlowJo analysis software, draw gates on the scatter plots to exclude cell debris and dead cells using the FSC-A and SSC-A.
  v. Draw gates to exclude doublet cells using the FSC-A and FSC-height (FSC-H) and gates to isolate the live population for subsequent analysis using the FSC-A and viability dye (AmCyan-A).
  w. Draw gates for each antibody using isotype controls.
  x. Generate scatter plots and histograms for each antibody, comparing the antibody staining to the isotype control to show the positively stained population (Figure 2).

**Figure 2:**
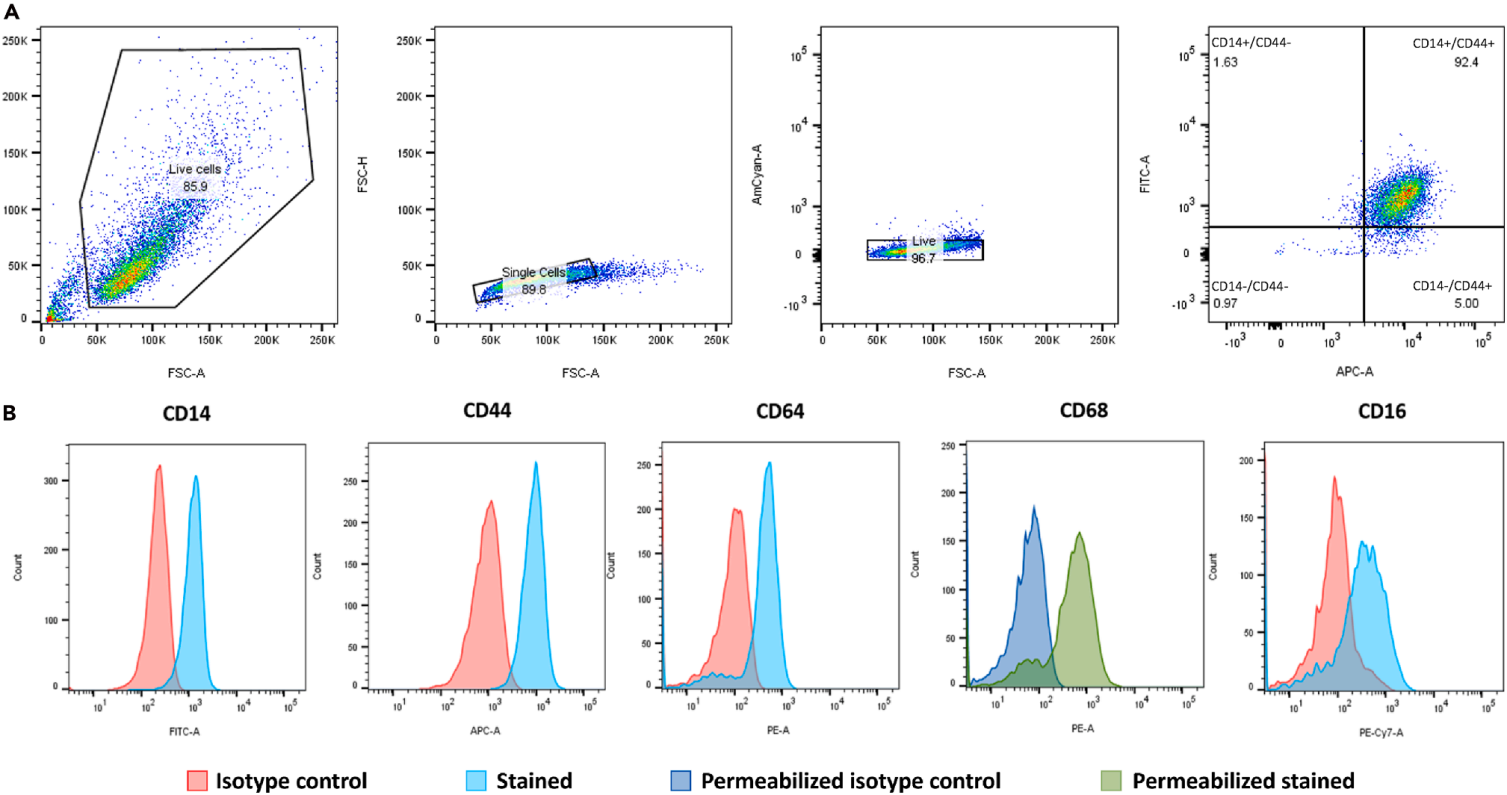
Macrophage marker staining of iMacs (A) Gating strategy used to identify iMacs and (B) representative histograms showing expression of macrophage markers CD14, CD44, CD64, CD68, and CD16 in unstimulated iMacs within the live and single cell population relative to the appropriate isotype control, *n* = 3 iMac differentiation batches.^1^
8. Phagocytosis assay.
  a. Seed iMacs at 1.25x10^5^ per well in 24 well plates on glass coverslips in iMac medium and incubate for up to 24 h.
  b. Remove RPMI and replace with 500 μL OptiMEM per well.
  c. Dilute latex amine-modified polystyrene, fluorescent yellow-green beads 1:10 in DPBS and add 10 μL per well.
  d. Incubate the iMacs with the fluorescent beads for either 30 or 90 min at 37°C.
  e. Remove the OptiMEM and wash the cells twice in 500 μl DPBS.
  f. Add 500 μl fresh OptiMEM with 0.5 μM LysoTracker Red DND-99 for 30 min at 37°C.
  g. Upon completion of the experiment, wash cells in 500 μl DPBS.
  h. Fix cells with 250 μl 4% paraformaldehyde (PFA) for 15 min before washing with DPBS.
    **Pause point:** Fixed coverslips can be stored in DPBS in the plates at 4°C for up to two weeks until ready to stain.
  i. Wash the coverslips 3 times in PBS.
  j. Remove coverslips from the 24 well plates and keep in a humidified chamber.
  k. Add 0.1% Triton X-100 in PBS for 10 min to permeabilize cells for staining.
  l. Wash the coverslips 3 times in PBS.
  m. Add 40 μl 50 mM ammonium chloride for 10 min to quench the PFA.
  n. Wash the coverslips 3 times in PBS.
  o. Block the coverslips with 0.5% BSA in PBS for 30 min.
  p. Wash the coverslips in PBS.
  q. Mount the coverslips in Vectashield antifade mounting medium with DAPI on glass microscope slides.
  r. Seal the mounted coverslips with clear nail polish and allow to dry.
    **Pause point:** Sealed coverslips can be stored at 4°C until ready for imaging.
  s. Image coverslips at 63 X (oil lens) using a confocal microscope and take images at a 16-bit resolution using a 1024x1024 scan format.
    ***Note:*** Image representative areas using Z-stack intervals of 0.3 μm to demonstrate the fluorescent beads are inside the lysosomes.
  t. Analyze the images using Fiji, count the number of DAPI+ cells and DAPI+bead+ cells manually.
  u. Calculate the percentage of DAPI+bead+ cells (Figure 3).

**Figure 3:**
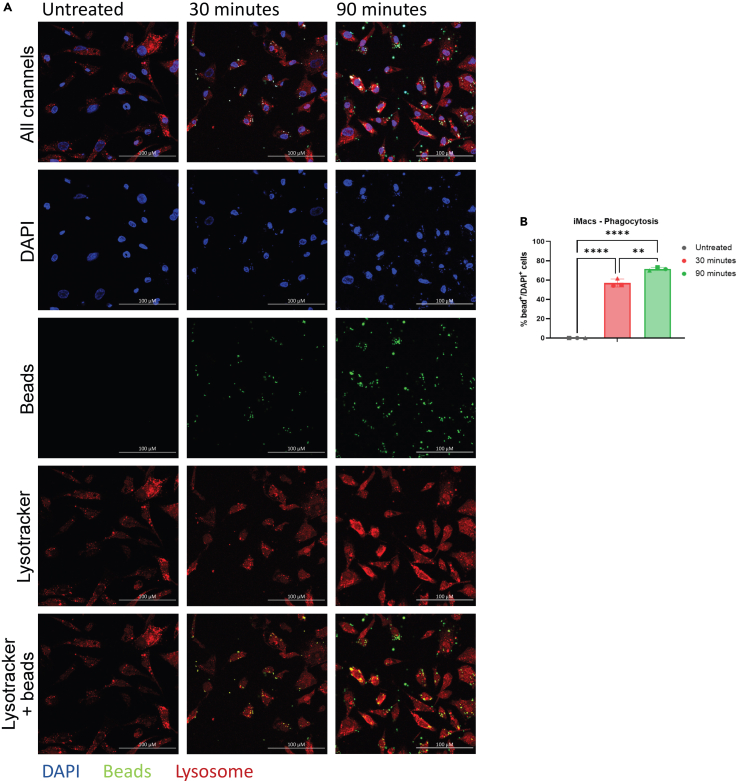
Phagocytic activity of iMacs (A) 63 X (oil lens) magnification Stellaris-5 confocal microscope representative images from iMacs in untreated cells and following incubation with Latex amine modified polystyrene fluorescent beads (green) for 30 min or 90 min stained with DAPI (blue) and Lysotracker (red). Scale bar = 100 μM. (B) Quantification of iMacs containing at least one fluorescent bead. Data are represented as mean ± SEM, each symbol represents an independent replicate, *n* = 3 independent replicates, ∗∗*p* < 0.01, ∗∗∗∗*p* < 0.0001 by One-way ANOVA with Tukey’s multiple comparisons test.^1^
9. Mass spectrometry.
  a. Seed iMacs at 1x10^6^ in 1 mL in 6 well plates and challenge with LPS (100 ng/ml) for 6-24 h.
  b. Wash the cells with ice-cold PBS, detach using cell scrapers, and collect into Eppendorfs.
  c. Centrifuge the cells at 13,000 × *g* at 4°C for 10 min.
  d. Remove the PBS supernatant and freeze the pellets at −80°C.
    **Pause point:** Cell pellets can be stored at −80°C until ready for sample preparation.
  e. Lyse cells with 200 μl of 1% SDC lysis buffer (1% SDC, 10 mM TCEP, 40 mM 2-chloroacetamide (CAA), 100 mM Tris pH 8.5).
  f. Heat the samples for 10 min at 95°C with shaking (800 rpm).
  g. Sonicate the samples for 2 min until the pellet is completely dissolved.
  h. Centrifuge the lysates at max speed (∼13,000 × *g*) for 15 min.
  i. Transfer the supernatants into a new tube and store at −20°C for 1-2 days until further processing.
  j. Use automated protein aggregation capture (PAC) digestion protocol adapted from^6^ using the Opentrons OT-2 liquid handling system to prepare samples for mass spectrometry.
  k. Add 8 μL of lysates and 5 μL of MagReSyn Hydroxyl beads (ReSyn Biosciences) in a final volume of 70% Acetonitrile (ACN).
  l. Incubate for 20 min to allow protein-bead aggregation, with one mixing step after 10 min.
  m. Wash the bead–protein aggregates twice with 140 μL of ACN and once with 170 μL of 50 mM triethylammonium bicarbonate buffer (TEAB) pH 8.5 at a slow speed (25 μl/second).
  n. For on-bead digestion, use 35 μl of digestion solution (50 mM TEAB) containing Lys-C and Trypsin at an enzyme-to-substrate ratio of 1:100 and 1:50, respectively for 4 h at ∼20°C.
  o. Activate Evotips (Evosep) by soaking in isopropanol for 10 s.
  p. Pre-condition Evotips by pushing through 20 μL of 0.1% Formic Acid (FA).
  q. Load 50% of the peptides onto Evotips.
  r. Acquire proteomics data on Mass spectrometer (MS).
    ***Note:*** We used a timsTOF HT (Bruker) quadrupole time-of-flight mass spectrometer integrating trapped Ion Mobility (IM) separations coupled online via a Captivespray electrospray source (Bruker) to Evosep One liquid chromatography system.^7^^,^^8^
  s. Separate peptides on an integrated packed emitter column (15 cm × 75 μm ID – Coann) packed with 1.5 μm Reprosil Saphir beads (Dr. Maisch) using whisper 40 SPD method.
  t. To acquire data, use a diaPASEF method consisting of 12 cycles including a total of 24 windows, covering m/z from 300 to 1400 Da with two mobility windows each, covering the ion mobility range (1/K0) from 0.70 to 1.42 V s/cm2.
  u. Keep the CaptiveSpray source at a capillary voltage of 1,600 V, with a dry gas flow rate of 3.0 L/min at 180°C.
  v. Set accumulation and ramp times to 100 ms, resulting in 180 ms per MS^1^ or MS^2^ (DIA-PASEF) scan, excluding transfer time.
  w. Program the collision energy as a function of ion mobility, following a straight line from 20 eV for 1/K0 of 0.6 V s/cm2 to 59 eV for 1/K0 of 1.6 V s/cm2.
  x. To calibrate TIMS, set elution voltage to linear mode to obtain 1/K0 ratios using three ions (m/z of 622, 922, 1222) from the ESI-L Tuning Mix (Agilent).
    ***Note:*** Optionally include additional gas phase fractionation runs (GPF) of a pooled sample to boost library coverage.^9^
10. Mass Spectrometry Data Analysis.
  a. Analyze mass spectrometry data in Spectronaut version 19.1 (Biognosys) using the using a direct DIA workflow with default BGS settings against UniProtKB human sequence database UP000005640 (downloaded January 2024 - 20,413 entries); specific Trypsin/P digestion, 2 missed cleavages, peptide length 7–52, carbamidomethyl cysteine set as fixed modification, oxidation of methionine and N-terminal methionine excision as variable modifications allowing up to five variable sites per peptide.
  b. For PSMs, peptides, and proteins, set the control the false discovery rate at 1%.
    ***Note:*** Precursor filtering should require a peptide–spectrum match q-value of ≤ 0.01 and a minimum posterior error probability of 0.2. Protein identifications should meet q-value thresholds of 0.01 for experiment-wide assessment and 0.05 for run-wide assessment.
  c. Perform protein grouping using the IDPicker algorithm.
  d. Perform protein quantification using the MaxLFQ algorithm on peak areas, followed by cross-run normalization, without imputation of missing values.
  e. Export the protein quantities table using a pivot report in Spectronaut.
  f. Generate downstream plots using R, using base R and the following packages: ggplot2, ggrepel, pheatmap (Figure 4).

**Figure 4:**
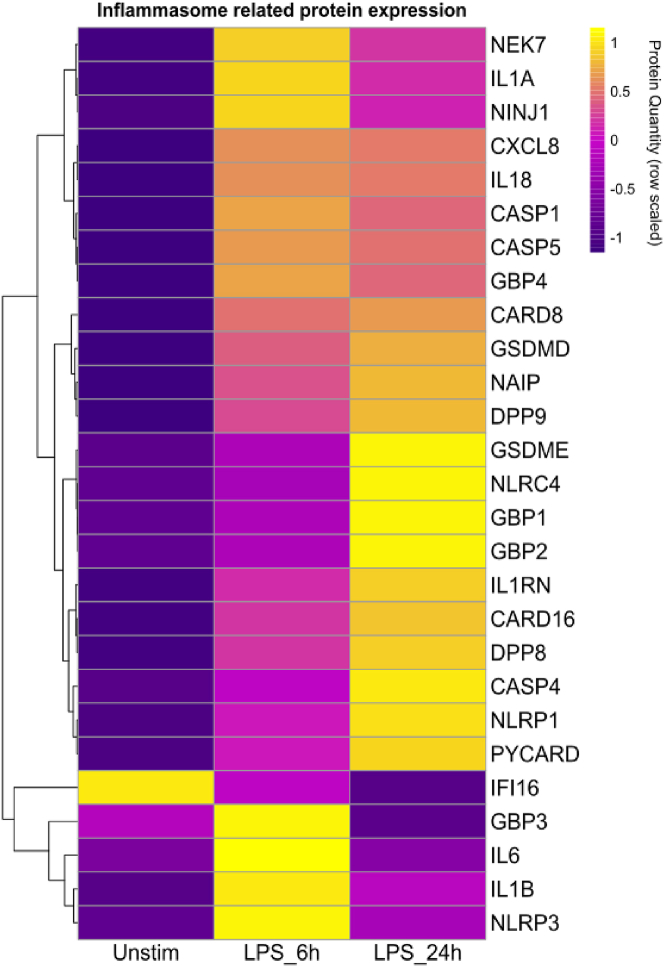
Expression of inflammasome-related proteins in iMacs Heatmap illustrating scaled protein quantity of inflammasome and inflammation related proteins (rows) following whole cell proteomics analysis of iMacs that were unstimulated or following stimulation with LPS (100 ng/ml) for 6 h or 24 h, *n* = 3 independent replicates. Clustering was performed using Euclidean distance and protein quantities are scaled by row.^1^

### Assessing inflammasome activity

**Timing: 1–2 weeks**

This step describes how to activate the NLRP3, non-canonical, NAIP/NLRC4, and NLRP1/CARD8 inflammasomes in iMacs. Inflammasome activity is subsequently assessed via measuring cell death (LDH) to indicate pyroptosis, cytokine release, expression and processing of inflammasome-related proteins via western blotting, and ASC speck formation via fluorescence microscopy.

11. Inflammasome activation.
  a. To activate NLRP3:
    i. Prime cells with 100 ng/mL ultrapure LPS from E. coli K12 or 1 μg/mL synthetic triacylated lipopeptide Pam3CSK4 for 4 h.
    ii. Activate with 5 μM nigericin sodium salt or 5 mM Adenosine 5-Triphosphate (ATP) disodium salt for 2 h.
  b. To activate the non-canonical inflammasome (NCI):
    i. Prime cells with 1 μg/mL Pam3CSK4 for 16 h.
    ii. Add fresh media.
    iii. Prepare LPS transfection mix – dilute 10 μL 1 mg/mL LPS in 87.5 μL OptiMEM (vortex vigorously) and add 2.5 μL Lipofectamine LTX Reagent (total LPS dilution 1:10)
    iv. Mix well and incubate at ∼20°C for 15 min.
    v. Prepare LPS alone control (LPS diluted 1:10 in OptiMEM).
    vi. Add LPS transfection mix or LPS control 1:10 per well (final LPS concentration = 10 μg/mL, 0.25% Lipofectamine LTX).
    vii. Centrifuge the plate at 1000 × *g* for 10 min at ∼20°C.
    viii. Incubate the plate for 5 h at 37°C, 5 % CO_2_.
  c. To activate NAIP/NLRC4, prime cells for 4 h and treat with 1 ng/mL Lfn-BsaL plus 250 ng/mL Anthrax Protective Antigen (PA) for 2 h.
  d. To activate NLRP1, prime cells for 4 h and treat with 10 μM Talabostat mesylate or 10 μM Anisomycin for 24 h.
12. Harvesting cells and supernatants for LDH assay, ELISA, and western blot.
  a. Plate 0.5x10^5^ cells in 100 μL in 96 well plates in iMac medium for up to 24 h.
  b. Replace medium with 100 μL OptiMEM per well.
    ***Note:*** Perform all inflammasome activation assays in OptiMEM medium as the reduced-serum formulation prevents serum proteins interfering with cytokine detection via ELISA.
  c. Add the indicated inflammasome priming/activation stimuli for times listed in section 11 a-d.
  d. Upon completion of experiment, add 2 μL 10% Triton X-100 lysis solution to the LDH 100% lysis control wells.
  e. Centrifuge the plates for 3 min at 500 × *g* at ∼20°C.
  f. Remove the cell free supernatants to be used to assess LDH release and perform ELISAs and harvest the cell lysates to perform Western blot.
13. LDH assay.
  a. Prepare LDH assay buffer – dilute catalyst 1:46 in dye solution.
  b. Add 25 μL supernatant to 25 μL LDH assay buffer in a transparent 96 well plate.
  c. Incubate the plate in the dark for 30 min at ∼20°C.
  d. Measure the absorbance at 490 nm using FluroStar plate reader.
  e. Calculate the percentage of cell death in each sample relative to the 100 % lysis (10% Triton X-100) control.
14. ELISA – follow protocol from manufacturer’s instructions (R&D IL-1β ELISA and TNF-α ELISA).
15. Western blot.
  a. Harvest cell lysates in 50 μL cell lysis buffer (66 mM Tris HCl pH 7.4% SDS) with 1:10,000 BaseMuncher.
    ***Note:*** Protein concentration can be quantified using the Pierce™ Rapid Gold BCA Protein Assay Kit according to manufacturer’s instructions to ensure equal protein loading.
  b. Add 1X NuPAGE LDS sample buffer plus 50 mM Dithiothreitol (DTT) to each sample.
  c. Boil samples for 5 min at 95°C and centrifuge for 1 minute at 1,000 × *g*.
  d. Add the Precision Plus Protein Kaleidoscope Standard to the first well (4 μL) of a 4–20% 10-well Mini-PROTEAN® TGX™ Precast Protein Gel in a BioRad western blot tank.
  e. Add up to 50 μL of prepared samples to the gel.
  f. Separate the proteins for 60 – 80 min at 120 V in 1X running buffer.
  g. Use a semi-dry protocol to transfer proteins from the gels to a nitrocellulose membrane.
  h. Layer a stack of filter paper, nitrocellulose membrane, the gel, and another stack of filter paper all soaked in transfer buffer onto the tray from the Trans-Blot® Turbo™ Transfer System.
  i. Select the turbo transfer, mixed MW, MIDI, 2.5A, 25V, 7 min program.
  j. After transferring, remove the membrane and wash in TBS-Tween.
  k. Block the membrane in 5% dried milk powder in TBS-Tween at ∼20°C for 1 h.
  l. Incubate the membrane in primary antibody in 5% milk in TBS-Tween for up to 24 h on a rocker at 4°C.
  m. The next day, wash the membranes 3 times for 10 min in TBS-Tween.
  n. Incubate in secondary antibody in 5% milk in TBS-Tween for 1 h at ∼20°C.
  o. Wash the membranes 3 times for 10 min in TBS-Tween.
  p. Add Clarity ECL for 1 minute.
    ***Note:*** For proteins that are difficult to detect, such as cleaved caspase-1, add SuperSignal™ West Atto 1:5 to the Clarity ECL.
  q. Image the fluorescence using the GeneSys imager.
  r. Deactivate the ECL using hydrogen peroxide and block the membrane with 5% milk in TBS-Tween before addition of a subsequent primary antibody.
16. ASC speck microscopy.
  a. Plate cells at 1x10^5^ per well in their culture medium for up to 24 h in 24 well plates with coverslips added.
  b. Replace medium with 500 μL OptiMEM per well.
  c. Add the indicated inflammasome priming/activation stimuli for the times listed in section 11 a-d.
  d. Wash cells in PBS and fix in 250 μL 4% PFA for 15 min.
  e. Store the coverslips in the plates at 4°C until ready to mount onto slides.
  f. Wash the coverslips 3 times in PBS.
  g. Remove coverslips from the 24 well plates and keep in a humidified chamber.
  h. Add 0.1% Triton X-100 in PBS for 10 min to permeabilize cells for staining.
  i. Wash the coverslips 3 times in PBS.
  j. Add 50 mM ammonium chloride for 10 min to quench the PFA.
  k. Wash the coverslips 3 times in PBS.
  l. Block the coverslips with 0.5% BSA in PBS for 30 min.
  m. Dilute the primary anti-ASC antibody 1 in 50 in 0.5% BSA/PBS and add for 60 min in the dark.
  n. Wash the coverslips in PBS 4 times for 5 min.
  o. Dilute the Alexa Fluor™ 488 Donkey anti-mouse 1 in 800 in 0.5% BSA/PBS and add for 60 min in the dark.
  p. Wash the coverslips in PBS 4 times for 5 min.
  q. Mount in mounting media containing DAPI on glass microscope slides.
  r. Seal the mounted coverslips with clear nail polish and allow to dry.
  s. Store at 4°C until imaging.
  t. Image coverslips using the Leica LAS X software and the DM5500 fluorescent microscope at 20X and 40X.
  u. Analyze six fields of view per condition in each experiment with 2-3 technical replicates (coverslips) and 3 independent experiments.
  v. Quantify ASC specks in 20X images using Fiji.
    ***Note:*** An automated system can used to quantify the number of DAPI+ cells – set the prominence to 20 and strict in find maxima.
  w. Manually count the number of ASC specks per image using the multipoint tool.
  x. Calculate the percentage of DAPI+ASC+ positive cells.

## Expected outcomes

Following the differentiation protocol outlined here, iMacs express high levels of CD14, CD44, CD64, and CD68 as detected by flow cytometry (Figure 2). Expression of CD16 is more intermediate compared to the other markers tested, with around 50% positive staining (Figure 2) as has been previously observed for iMacs.^4^^,^^10^ In addition, iMacs are highly phagocytic with around 50% of cells phagocytosing at least one fluorescent bead after 30 min, which is further increased to around 70% by 90 min, with many of the cells containing multiple beads (Figures 3A and 3B). iMacs also highly express inflammasome sensors, such as NLRP3 and NLRC4, and other inflammasome components such as ASC, caspase-1, and GSDMD (Figure 4). In addition, LPS stimulation increases expression of inflammasome components NLRP3 and pro-IL-1β and inflammasome-independent cytokines, such as IL-6 and CXCL8 (Figure 4). As expected, expression of these nuclear factor κB (NF-κB)-dependent proteins peaks at 6h LPS and decreases by 24h (Figure 4). This is consistent with previous findings in primary macrophages and reflects the requirement for rapid responses to bacterial stimulation to clear infection and later resolution to limit damaging inflammation.^11^ Interestingly, expression of other proteins, such as caspase-4 and NLRC4, increases later following 24h LPS stimulation (Figure 4). Prolonged LPS exposure (24h) may be required to trigger other transcription factors, such as interferon regulatory factors (IRFs), to regulate expression of these proteins. Future work could explore these patterns of protein expression further.

**Figure 5:**
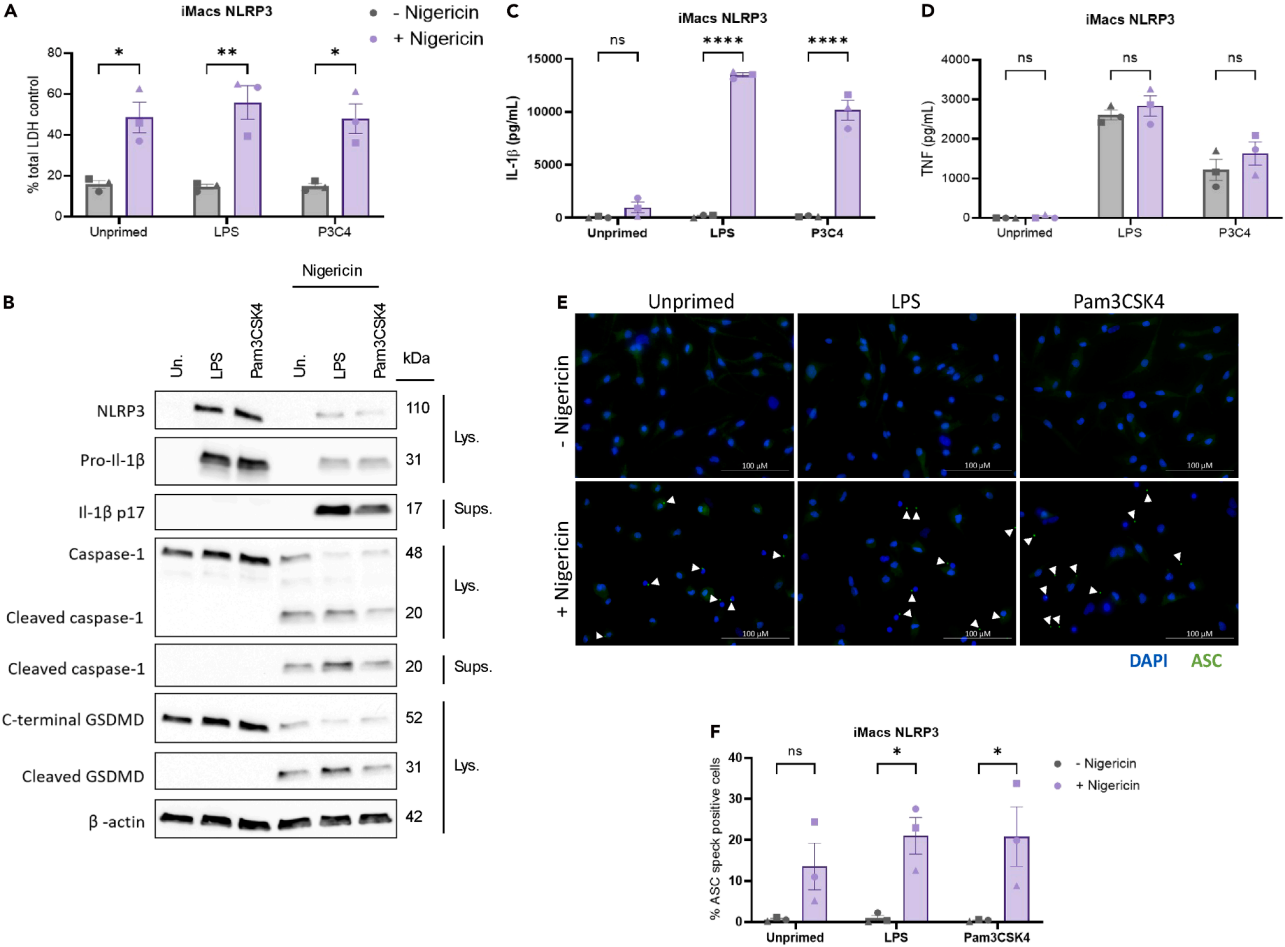
NLRP3 activation in iMacs (A) LDH release, (B) Western blots, (C) IL-1β release, and (D) TNF release from iMacs in unprimed cells and following stimulation with 100 ng/ml LPS or 1 μg/ml Pam3CSK4 for 4 h with 5 μM nigericin for 2 h. (E) Representative images from iMacs in unprimed cells and following stimulation with 100 ng/ml LPS or 1 μg/ml Pam3CSK4 for 4 h with 5 μM nigericin for 2 h stained with DAPI (blue) and ASC (green) with ASC specks highlighted with white arrows. Scale bar = 100 μM. (F) Quantification of ASC speck positive cells. Data are represented as mean ± SEM, each symbol represents an independent replicate, *n* = 3 independent replicates, ∗*p* < 0.05, ∗∗*p* < 0.01, ∗∗∗∗*p* < 0.0001 by Two-way ANOVA with Šídák’s multiple comparisons test.^1^

iMacs are also highly responsive to NLRP3 inflammasome activation stimuli. Treatment with nigericin causes significant inflammasome-dependent cell death with LDH release and cleavage of both caspase-1 and GSDMD (Figure 5 A, B). Inflammasome-dependent cell death is seen even in unprimed cells suggesting that, unlike primary human macrophages,^1^ iMacs can respond to nigericin alone. Priming with LPS or Pam3CSK4 upregulates expression of pro-IL-1β and NLRP3 (Figure 5B) and subsequent activation with nigericin triggers significant mature IL-1β release (Figure 5C). In addition, priming with LPS or Pam3CSK4 triggers release of the inflammasome-independent cytokine TNF (Figure 5D). High levels of cytokine release are seen in response to these bacterial stimuli in the iMacs, consistent with findings in primary human macrophages.^1^^,^^11^ This illustrates the requirement for rapid and robust immune responses to microbial stimuli to limit pathogen dissemination.^11^ As an additional readout of inflammasome activation, LPS or Pam3CSK4 priming plus nigericin triggers significant ASC speck formation (Figures 5E and 5F). Therefore, the NLRP3 inflammasome is highly responsive to priming and activation stimuli in iMacs. The responses of iMacs to other inflammasome activation stimuli, including non-canonical, NAIP/NLRC4, and NLRP1, are outlined in McKee et al.^1^

Overall, iMacs express high levels of macrophage markers and inflammasome-related proteins, are highly phagocytic, and respond robustly to inflammasome activation. As a result, iMacs are a physiologically relevant model to study the activation and regulation of inflammasomes in human macrophages. Unlike primary human macrophages, iMacs can easily be genetically modified, therefore opening up a wide range of new opportunities to study inflammasome activation in macrophages.

## Limitations

The genetic background of the iPSC donor used to generate the iMacs must be considered as mutations could influence the results. Also, iMacs are embryologically more similar to tissue-resident macrophages than monocyte-derived but to study these cells as tissue-resident, further differentiation is required into terminally differentiated macrophages, such as microglia or alveolar macrophages.

## Troubleshooting

### Problem 1

EBs form normally and adhere to gelatine-coated flasks but do not produce any myeloid precursors (steps 4 and 5).

### Potential solution

The differentiation potential can vary between iPSC lines for different cell types therefore it may be necessary to use a different iPSC line. Ensure that the iPSC line selected has successfully been differentiated into iMacs previously, such as the kolf_2 cells used here or the ieki_2 cells used in McKee et al.^1^

### Problem 2

EBs produce myeloid precursor cells but the yield is very low (step 5).

### Potential solution

Adapt the myeloid precursor generation stage (section 5d-f) to increase the number of embryoid bodies per flask or the number of flasks prepared. Also consider adapting the iPSC number used to generate the embryoid bodies as the optimal number may vary between iPSC lines.

### Problem 3

Low staining detected for macrophage markers via flow cytometry (step 7).

### Potential solution

Optimize the antibody concentration used or use a positive control, such as human monocyte-derived macrophages to compare levels of staining.

## Resource availability

### Lead contact

Further information and requests for resources and reagents should be directed to and will be fulfilled by the lead contact, Dr. Rebecca C. Coll (r.coll@qub.ac.uk).

### Technical contact

For any technical questions regarding this protocol, please contact Chloe M. McKee (chloe.mckee@qub.ac.uk).

### Materials availability

Any material and information used for this study is available from the lead contact upon request.

### Data and code availability

• The mass spectrometry proteomics data have been deposited to the ProteomeXchange Consortium via the PRIDE partner repository^12^ (https://www.ebi.ac.uk/pride/login) with the dataset identifier PRIDE: PXD068076, token: ilXveQUXDATv.
• This study does not report original code.

## Acknowledgments

This work was supported by funding from the 10.13039/501100000691Academy of Medical Sciences (SBR005\1104) (to R.C.C.), 10.13039/501100000268Biotechnology and Biological Sciences Research Council (BB/V016741/1) (to R.C.C. and B.C.C.), and Northern Ireland Department of the Economy PhD studentships (to C.M.M. and E.C.M.). We would like to thank Dr. Subhankar Mukhopadhyay (King’s college London) for helpful discussions about iPSC-derived macrophage culture. We would also like to acknowledge the flow cytometry core technical unit at Queen’s University Belfast.

## Author contributions

Conceptualization, R.C.C. and C.M.M.; funding acquisition, R.C.C. and B.C.C.; investigation, C.M.M., M.C., E.C.M., and M.A.; formal analysis, C.M.M., E.C.M., M.A., and B.C.C.; project administration, R.C.C.; supervision, R.C.C. and B.C.C.; visualization, C.M.M. and E.C.M.; writing – original draft, R.C.C. and C.M.M.; writing – review and editing, C.M.M., M.C., E.C.M., M.A., B.C.C., and R.C.C.

## Declaration of interests

R.C.C. is a co-inventor on patents and patent applications for NLRP3 inhibitors, which have been licensed to Inflazome Ltd., Ireland. R.C.C. is a consultant for BioAge Labs, USA (since 2020), and serves on the Scientific Advisory Board of Viva in Vitro Diagnostics, Spain (since 2024). B.C.C. receives or has received research funding from Bruker Daltonics and Almac Discovery.
